# Ballistic Performance of Nanostructured Metals Toughened by Elliptical Coarse-Grained Inclusions: A Finite Element Study with Failure Analysis

**DOI:** 10.3390/ma11060977

**Published:** 2018-06-08

**Authors:** Xiang Guo, Qidong Ouyang, Yubo Sun, George J. Weng

**Affiliations:** 1 School of Mechanical Engineering, Tianjin University, Tianjin 300072, China; sunyubo@tju.edu.cn; 2 Tianjin Key Laboratory of Nonlinear Dynamics and Control, Tianjin 300072, China; 3 State Key Laboratory for Strength and Vibration of Mechanical Structures, Xi’an Jiaotong University, Xi’an 710049, China; 4 Jiangsu Testing Center for Quality of Construction Engineering Co., Ltd., Nanjing 210028, China; oyqd@foxmail.com; 5 Department of Mechanical and Aerospace Engineering, Rutgers University, New Brunswick, NJ 08903, USA; gjweng@soe.rutgers.edu

**Keywords:** bimodal nanostructured metals, ballistic performance, Johnson-Cook failure criterion, microstructure, elliptical inclusions

## Abstract

Bimodal nanostructured (NS) metals, in which the nano-grains or ultrafine grains serve as matrix phase while the coarse grains serve as toughening phase, can synergize the overall strength and ductility to achieve excellent bullet-proof performance. Because of the extrusion process in the fabrication, the coarse-grained (CG) inclusions are elongated in the extrusion direction and elliptical CG inclusions with different aspect ratios form. The shape, distribution, and volume fraction of these elliptical CG inclusions can all have significant influence on the overall ballistic performance. In this study, the strain gradient plasticity model together with the Johnson–Cook failure criterion is employed to investigate the ballistic performance of the bimodal NS Cu with elliptical CG inclusions. Our results show that the ballistic performance can be improved by increasing the aspect ratio of the elliptical CG inclusions. Furthermore, the staggered distribution of the elliptical CG inclusions will decrease the overall ability of the material to resist failure, but it will improve its overall ability to resist deformation. The larger stagger degree of elliptical CG inclusions can weaken their shape effects on the limit displacement.

## 1. Introduction

With the improvement of technology in weaponry manufacturing, the power of firearms against the target has increased tremendously. The traditional armor or helmet is less likely to provide effective security for the safety of soldiers and military facilities. Therefore, many researchers are now focusing on the design of new microstructures to obtain superior bullet-proof materials with a higher strength and ductility.

Bimodal nanostructured (NS) metals are a new kind of metals that could serve this function. Different from traditional two-phase materials where the second phase with different composition is embedded into the matrix phase to play a strengthening or toughening role, two phases in the bimodal NS metals have the same composition but different grain sizes. This class of metals can be regarded as a “composite” where the coarse-grained (CG) inclusions are distributed in the nano-grained (NG) matrix or “single-phase metals with local variations in the grain size”. The NG phase provides the required high strength, while the CG phase offers the required high ductility for the composite. The bimodal distribution makes a good use of the advantages of each phase and effectively solves the dilemma that high strength and good ductility are difficult to coexist as in traditional metals [1]. Early in 2001, the concept of bimodal composites was proposed in the study of NS Al–Mg alloy by Tellkamp et al. [2,3]. Different from traditional alloys, this composite metal is the first kind of composite with a bimodal distribution of grain size. Subsequently, more kinds of bimodal composites, such as bimodal NS Cu–Al alloy fabricated by Lau et al. [4], have been obtained.

Extensive experimental study has shown that bimodal NS metals have superb combination of strength and ductility [5,6,7,8,9,10,11,12,13,14,15]. An extrinsic mechanism (crack blunting, crack bridging, and debonding [2,16,17,18,19,20,21]) and an intrinsic one (strain hardening of the CG phase [2,22,23,24,25]) contribute to their enhanced ductility. The strength and ductility of the bimodal NS Cu were found to be closely related to the volume fraction of the two phases, which makes it possible to improve the mechanical properties of the bimodal NS metals by tailoring the fabrication process [26].

According to the deformation mechanism of the bimodal NS metals, it is possible to describe their mechanical properties by various micromechanics models. For instance, Joshi et al. employed the secant Mori-Tanaka mean-field approach to obtain the elasto-plastic response of bimodal NS metals [27]. Self-consistent scheme in conjunction with the “translated fields” technique can also be used to calculate their visco-elasto-plastic response as a function of grain size distribution in bimodal metals [28]. However, all of these models cannot address the issues of crack initiation, propagation, and fracture of the two-phase solid.

The ballistic responses of bimodal NS metals involve all of these deformation and failure processes. Due to the technological importance of ballistic impact, this problem has attracted wide attention in the research community. Experimental results showed that bimodal ultrafine-grained Al plate with a thickness 13 mm was equivalent to standard Al plate with a thickness 19 mm in limit velocity and it saved 33% weight [18]. The effects of the microstructures of the bimodal Ti alloy on the overall ballistic performance were investigated by Zheng et al. It has been disclosed that, as the microstructure varies, so does the fracture mechanism as well as the type of shear bands [29]. To provide more comprehensive information, numerical simulations have also been conducted on bimodal NS metals [30,31,32,33,34]. One important outcome is that, by microstructural design, the bimodal NS metals can effectively synergize high strength and good ductility to improve their bullet-proof performance [32,34].

The bimodal NS metals can be fabricated by blending the cryomilled NG powders with CG powder [21]. The powder blends are canned and then consolidated by pressing at a certain pressure. Afterwards, the consolidated compacts are degassed at a certain temperature. To remove remaining porosity, the consolidated billets are then extruded. The extrusion process leads the CG inclusions to become elongated in the extrusion direction and elliptical in shape [7,13,17,21,35,36]. They have different ratios of major to minor axis, known as the aspect ratio of inclusions, and also different distributions, characterized by the stagger degree. Therefore, the study on the ballistic performance of the bimodal NS metals with elliptical CG inclusions is directly linked to the real microstructures. However, this problem has never been taken up anywhere to date.

This is the objective of this investigation, and this investigation is the first systematic trial using the numerical approach. In the present study, the strain gradient plasticity model together with the Johnson–Cook failure criterion will be employed to simulate the ballistic performance of the bimodal NS Cu with elliptical CG inclusions. Our focus will be on the effects of shape, distribution, and volume fraction of the elliptical CG inclusions on the overall ballistic performance that covers both strength and ductility.

## 2. Modelling Method

### 2.1. Idealized Microstructures

**Figure 1:**
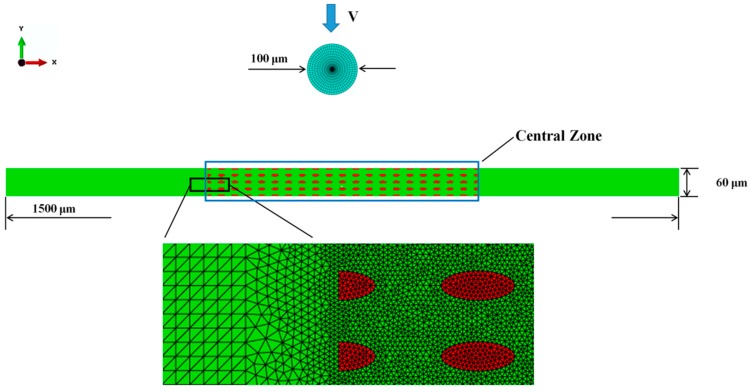
Specimen configuration.

A bimodal NS Cu specimen with dimensions 1.5 × 0.06 mm^2^ in the X-Y plane is subjected to a ballistic impact, as depicted in Figure 1. Specifically, a bullet with a diameter 0.1 mm impacts the central zone of the specimen. The central zone is idealized as a microstructure with dimensions 0.6 × 0.06 mm^2^ and the two end zones as a homogenized phase. To seek a balance between computational accuracy and efficiency, linear triangular elements [37] with feature size 1 µm and 3 µm are used in the central zone and the end zones, respectively. Linear elements [37] with a feature size 5 µm are used for the bullet. The finite element meshes in the central, the transitional, and the end zones of a sampled specimen, together with those in the bullet, are also shown in Figure 1. The left edge of the specimen is fixed; the right one is also fixed except that its movement along the horizontal direction is free. In addition, an initial temperature field 25 °C is applied. The condition of plane strain is assumed. Our dynamic simulation is conducted in ABAQUS [37].

**Figure 2:**
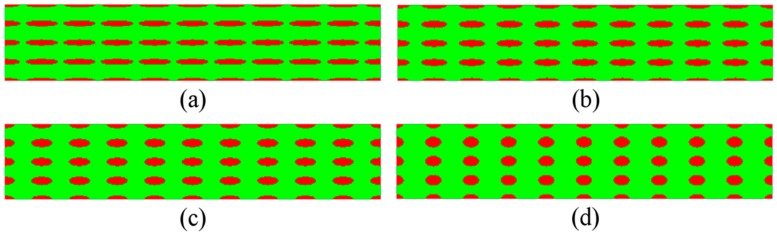
Microstructures with different *size*: (**a**) 2 × 12.5; (**b**) 2.5 × 10; (**c**) 3.125 × 8; and (**d**) 4 × 6.25 μm.

**Figure 3:**
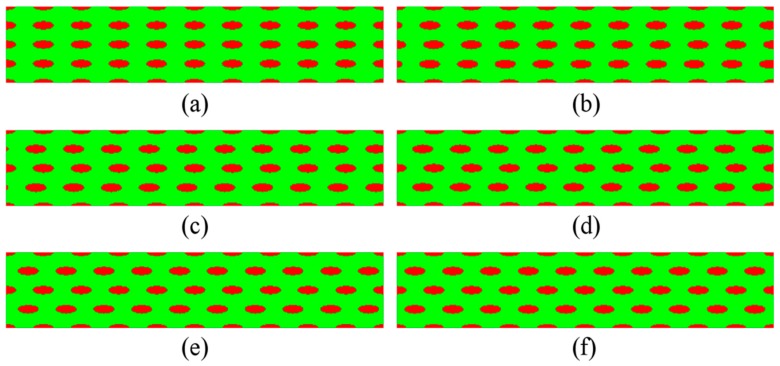
Microstructures with the same *size* 3.125 × 8 μm and different *stagger degree*: (**a**) 0%; (**b**) 10%; (**c**) 20%; (**d**) 30%; (**e**) 40%; and (**f**) 50%.

**Figure 4:**
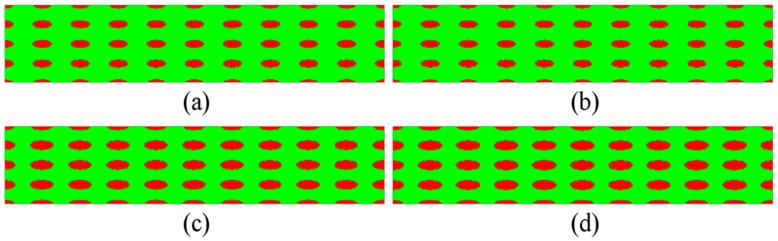
Microstructures with the *f*: (**a**) 14.13%; (**b**) 17.45%; (**c**) 21.11%; and (**d**) 23.07%.

Based on the numerical scheme of Guo et al. [30,31,32,33,34] on bimodal NS metals and the design approach on the two-phase microstructures, the elliptical CG inclusions are systematically introduced into this study. Since aspect ratio and volume fraction of the inclusions significantly affect the overall elasto-plastic properties and behavior of the composites with spheroidal inclusions [38,39,40,41], several series of microstructures are designed by using elliptical CG inclusions with different shape, distribution, and volume fraction, as depicted in Figure 2, Figure 3 and Figure 4 with the NG phase in green and the CG phase in red.

Figure 2 illustrates the four microstructures with array-arranged CG inclusions. While the volume fraction of CG inclusions (*f*) is the same (~17.45%), the lengths of half-minor and half-major axis of CG inclusions are 2 × 12.5, 2.5 × 10, 3.125 × 8, and 4 × 6.25 μm in Figure 2a–d, respectively. To facilitate the description, we refer to the length of half-minor and half-major axis of CG inclusions in the microstructures as *size* for short. The aspect ratios of the four microstructures are 6.25, 4, 2.56, and 1.5625 in Figure 2a–d, respectively. The larger the aspect ratio is, the more flattened the shape of the CG inclusions is.

Via displacing the second and fourth layers of CG inclusions in the microstructures from Figure 2 horizontally towards the left, we get microstructures with staggered CG inclusions. We define the stagger degree of CG inclusions as the ratio of displacement of the second and fourth layers of CG inclusions to the central distance of two horizontally adjacent inclusions. Figure 3 shows six microstructures with CG inclusions in different stagger degree when the *size* is 3.125 × 8 μm and the *f* is 17.45%. Similarly, when the *sizes* are 2 × 12.5, 2.5 × 10, and 4 × 6.25 μm, other eighteen microstructures with CG inclusions in different stagger degree can also be designed. To facilitate the description, we refer to the stagger degree of CG inclusions in a microstructure as the *stagger degree* for brevity.

To investigate the effects of volume fraction of CG inclusions on the overall ballistic performance, we design sixteen microstructures with different *f* based on the four microstructures in Figure 2. In Figure 4b, the microstructure is the same with that in Figure 2c, whose *size* is 3.125 × 8 μm and the *f* is 17.45%. The sizes of two axes of CG inclusions in Figure 4a are decreased by 10% relative to those in Figure 4b and the *f* is 14.13%. The sizes of two axes of CG inclusions in Figure 4c and d are enlarged 10% and 15% relative to those in Figure 4b and the *f* is 21.11% and 23.07%, respectively.

Similarly, based on the microstructures with the *size* 2 × 12.5, 2.5 × 10, and 4 × 6.25 μm in Figure 2, other twelve microstructures with different *f* can also be designed. Table 1 shows the actual *size* of sixteen microstructures. We call 2 × 12.5 μm the base *size* of microstructures in Group I, and 2.5 × 10, 3.125 × 8, and 4 × 6.25 μm the base *size* of microstructures in Group II, III, and IV. Every four microstructures in the same group have the same aspect ratio.

### 2.2. Constitutive Relation and Failure Criterion of the NG Phase

In the NG phase, the effects of grain boundaries should be carefully considered because of their increased volume concentration. The geometrically necessary dislocations pile up along them. Due to the dislocation pileup zones near the grain boundaries with prominent strain gradients, the conventional theory of mechanism-based strain gradient plasticity has been used to deal with the contribution of the geometrically necessary dislocations [42].

The strain rate tensor $\dot{\varepsilon }$ has elastic and plastic parts:

(1) $$\dot{\varepsilon }={\dot{\varepsilon }}^{e}+{\dot{\varepsilon }}^{p}$$

${\dot{\varepsilon }}^{e}$ can be formulated by the elastic compliance tensor M and the stress rate tensor $\dot{\sigma }$ into

(2) $${\dot{\varepsilon }}^{e}=M:\dot{\sigma }.$$

According to the *J*_2_-plasticity flow rule, ${\dot{\varepsilon }}^{p}$ is proportional to the deviatoric stress $\sigma^{\prime }$:

(3) $${\dot{\varepsilon }}^{p}=\frac{3{\dot{\varepsilon }}_{e}^{p}}{2\sigma_{e}}\sigma^{\prime }$$

where $\sigma_{ij}^{\prime }=\sigma_{ij}-\sigma_{kk}\delta_{ij}/3$ (i,j=1,2,3), $\sigma_{e}=\sqrt{3\sigma_{ij}^{\prime }\sigma_{ij}^{\prime }/2}$ is von Mises stress, and ${\dot{\varepsilon }}_{e}^{p}=\sqrt{2{\dot{\varepsilon }}_{ij}^{p}{\dot{\varepsilon }}_{ij}^{p}/3}$ the equivalent plastic strain rate. A power law can be used to describe ${\dot{\varepsilon }}_{e}^{p}$ as [43]

(4) $${\dot{\varepsilon }}_{e}^{p}{=\dot{\varepsilon }}_{e}{\left[\frac{\sigma_{e}}{\sigma_{\mathrm{flow}}}\right]}^{m_{0}}$$

where ${\dot{\varepsilon }}_{e}=\sqrt{2{\dot{\varepsilon }}_{ij}^{\prime }{\dot{\varepsilon }}_{ij}^{\prime }/3}$ is the equivalent strain rate, ${\dot{\varepsilon }}_{ij}^{\prime }={\dot{\varepsilon }}_{ij}-{\dot{\varepsilon }}_{kk}\delta_{ij}/3$, $m_{0}$ is the strain rate-sensitivity parameter, and $\sigma_{\mathrm{flow}}$ the flow stress of the NG phase.

Taylor’s model can relate the flow stress of the NG metal to the dislocation density due to dislocations in the nano-grain interior and that due to the grain boundary dislocation pileup zones [42]. The detailed formulations of flow stress together with the model parameters can be found in our former paper [31]. Furthermore, Young’s modulus of the NG and CG Cu can be taken as 124 GPa [44] and Poisson’s ratio of both phases as 0.34.

Johnson and Cook established a relation between stress versus strain, strain rate, and temperature (*T*) of metals at high strain rates [45], i.e.,

(5) $$\sigma_{e}=\left[A+B{\left(\varepsilon_{e}^{p}\right)}^{n}\right]\left[1+C\ln\left(\frac{{\dot{\varepsilon }}_{e}^{p}}{{\dot{\varepsilon }}_{0}}\right)\right]\left[1-{\left(\frac{T-T_{r}}{T_{m}-T_{r}}\right)}^{m}\right]\;(T_{r}\leq T\leq T_{m})$$

where *A*, *B*, *C* (0.05 for NG Cu and 0.025 for CG Cu), *m* (1.09), and *n* are model parameters, ${\dot{\varepsilon }}_{0}$ (1 s^−1^) a reference strain rate, $T_{r}$ (25 °C) the room temperature, and $T_{m}$ (1083 °C) the melting temperature of Cu. With the above calibrated strain gradient plasticity model, we can fit the constitutive parameters of the NG Cu with the nano-grain size 23 nm as A=669 MPa, B=912 MPa, n=0.28. On the other hand, for the CG Cu, A=90 MPa, B=292 MPa, n=0.31.

Furthermore, Johnson and Cook proposed a criterion between a damage index *D* and the equivalent plastic strain increment ${d\varepsilon }_{e}^{p}$ in a linear fashion [46], i.e.,

(6) $$D=\int \frac{1}{\varepsilon_{f}}{d\varepsilon }_{e}^{p}=\int {\left\{\left[d_{1}+d_{2}e^{d_{3}\frac{p}{\sigma_{e}}}\right]\left[1+d_{4}\ln\left(\frac{{\dot{\varepsilon }}_{e}^{p}}{{\dot{\varepsilon }}_{0}}\right)\right]\left[1+d_{5}\frac{T-T_{r}}{T_{m}-T_{r}}\right]\right\}}^{-1}{d\varepsilon }_{e}^{p}$$

with $d_{1}$ to $d_{5}$ material constants and p the hydrostatic pressure. $d_{2}=d_{3}=0$ is taken as a good approximation in this study. Therefore, $d_{1}$ in Equation (6) can be interpreted as the failure strain at the ${\dot{\varepsilon }}_{0}$ and under the $T_{r}$. Therefore, for the NG Cu with the nano-grain size 23 nm, $d_{1}$ = 0.13 [47]. On the other hand, for the CG Cu, $d_{1}$ = 0.54. For both phases, $d_{4}$ = 0.014 and $d_{5}$ = 1.12. This criterion is used to investigate the failure process of the CG and NG phases. Their deformation at high strain rates is assumed to be adiabatic.

## 3. Results and Discussion

We put the thirty-six microstructures designed in Section 2.1 and constitutive relations elaborated in Section 2.2 into our finite element scheme to investigate the influence of shape, distribution, and volume fraction of elliptical CG inclusions (*size*, *stagger degree*, and *f*) on the overall ballistic performance and the intrinsic mechanisms of bimodal NS Cu. We analyze the ballistic performance by two indexes—(Ballistic) limit velocity *V*_b_ and (ballistic) limit displacement *D*_b_.

### 3.1. General Impact Process

The limit velocity *V*_b_ is defined as the lowest impact velocity with which the bullet fails the entire specimen, i.e., the bullet initiates microcracks to penetrate the entire specimen [48,49]. Higher *V*_b_ means stronger ability to resist failure under the ballistic impact.

**Figure 5:**
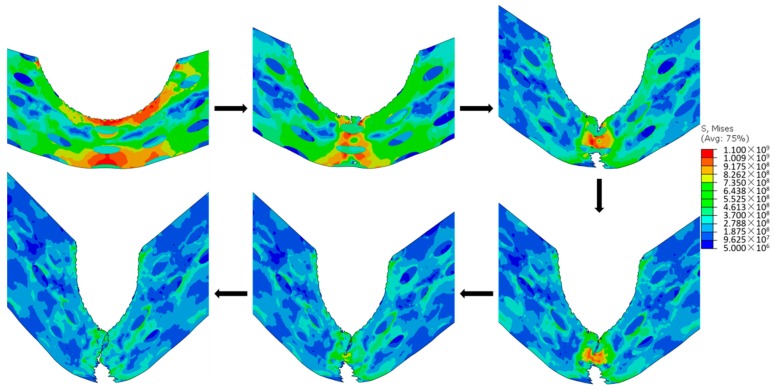
Failure process (stress in Pa).

By comparing the impact response of different microstructures at their own *V*_b_, the general failure process of various microstructures can be summarized. Figure 5 illustrates the failure process of a microstructure with the *f* 17.45%, the *size* 2.5 × 10 μm, and the *stagger degree* 0 at its *V*_b_. First, once the bullet touches the microstructure, the top of the microstructure including the upper two layers of CG inclusions are eroded instantaneously. Subsequently, the central zone of the microstructure moves downward and microcracks initiate at the top of the central zone and the top of the bottom layer of CG inclusions. Next, the upper microcracks propagate to the third layer of CG inclusions. Because of the tensile stress on the bottom of the microstructure initiated by the overall downward movement, the lower microcracks propagate upward and downward simultaneously, and penetrate the fifth layer of CG inclusions and propagate into the fourth layer. The upper and lower microcracks propagate, coalescence, and then fail the microstructure. In all of the failure processes of different microstructures, after the instantaneous erosion, the microcracks generally propagate around the central axis of the microstructure, and the region near the central axis bears most of the intensive stress during the impact process.

To investigate the deformation resistance of the microstructures, we let the bullet impact all microstructures at a fixed initial velocity 270 m/s. As it is lower than *V*_b_ of all thirty-six microstructures, they will not fail after the impact. The central zone of the specimen is hit directly and thus has a displacement. We define the maximum of the absolute value of this displacement as limit displacement—*D*_b_ [48,49]. Obviously, the smaller the *D*_b_ is, the smaller deformation the specimen has and the larger its deformation resistance is.

The bullet first impacts the specimen in a short duration and then it obtains a high initial velocity. Almost at the same time, the bullet velocity decreases dramatically, leading to the separation of the bullet and the microstructure. Subsequently, the microstructure rebounds up and down, and the amplitude of the rebound decreases with the dissipation of energy. The *D*_b_ occurs before the first rebound of the microstructure.

### 3.2. Effects of the Size of Elliptical CG Inclusions

**Figure 6:**
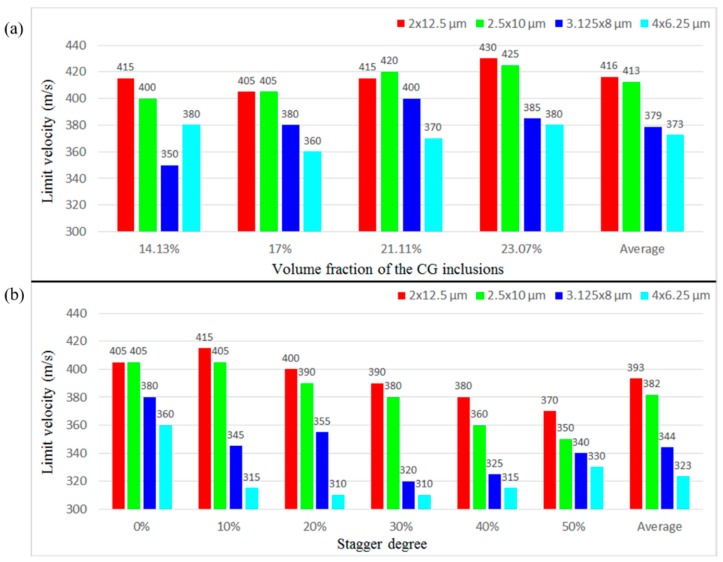
*V*_b_ of the microstructures with different *sizes* when (**a**) the *f* is fixed at different levels and the *stagger degree* is 0; and (**b**) the *stagger degree* is fixed at different levels and the *f* is 17.45%.

Figure 6a illustrates *V*_b_ of the microstructures with different *sizes* when the *f* is fixed at different levels and the *stagger degree* is 0. Figure 6b shows *V*_b_ of the microstructures with different *sizes* when the *stagger degree* is fixed at different levels and the *f* is 17.45%. We can see that when the *f* or the *stagger degree* is the same, except the case of *f* = 14.13%, the *V*_b_ decreases with the decrease in the aspect ratio, which indicates their decreasing ability to resist failure. The averaged *V*_b_ of microstructures with the same aspect ratio also reflects the similar trend, i.e., it decreases with the decrease in the aspect ratio. The results demonstrate that the larger the aspect ratio is, the higher the *V*_b_ of the microstructures is.

**Figure 7:**
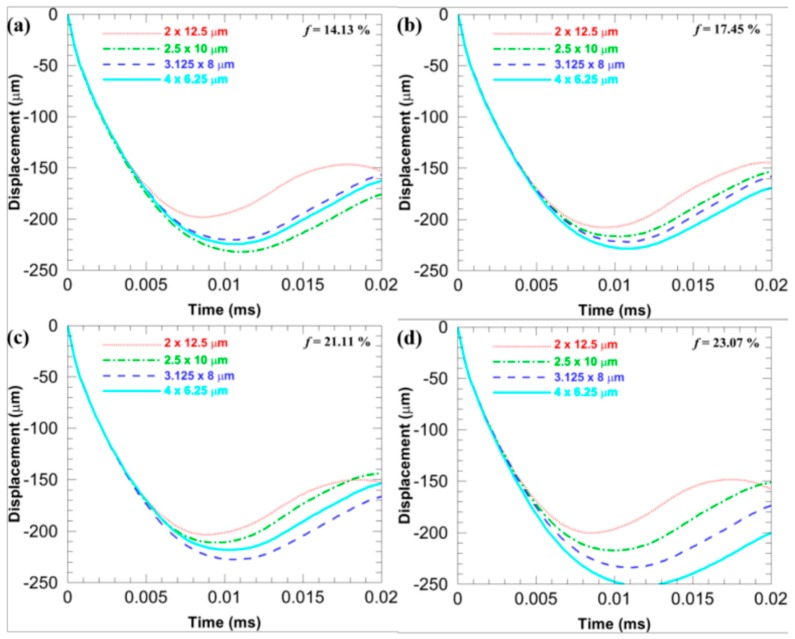
*D*_b_ of the microstructures when the *stagger degree* is 0 and the *f* is fixed at different levels: (**a**) 14.13%; (**b**) 17.45%; (**c**) 21.11%, and (**d**) 23.07%.

**Figure 8:**
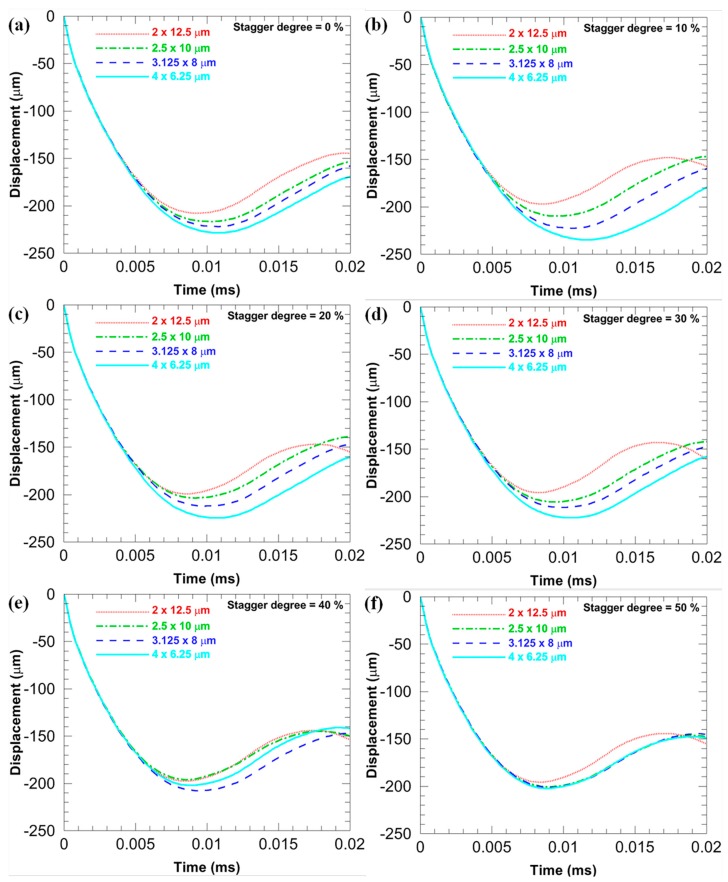
*D*_b_ of the microstructures with different *sizes* when the *f* is 17.45% and the *stagger degree* is fixed at different levels: (**a**) 0%; (**b**) 10%; (**c**) 20%; (**d**) 30%; (**e**) 40%; and (**f**) 50%.

Figure 7 compares *D*_b_ of the microstructures with different *sizes* when the *f* is fixed at different levels and the *stagger degree* is 0. Figure 8 compares *D*_b_ of the microstructures with different *sizes* when the *stagger degree* is fixed at different levels and the *f* is 17.45%. In Figure 7, when the *f* is the same, the more flattened the CG inclusions is, the smaller the *D*_b_ is (note that the *D*_b_ is an absolute value), which infers the larger deformation resistance. Similarly, when the *stagger degree* is the same, the more flattened the CG inclusions is, the smaller the *D*_b_ is, as shown in Figure 8. Furthermore, the *D*_b_ of the microstructures with different *sizes* are closer when the *stagger degree* is larger, which means the larger *stagger degree* can weaken the shape effects of CG inclusions on the *D*_b_.

As the aspect ratio increases, the microstructures exhibit the similar influence on *V*_b_ and *D*_b_—With the same *f* and *stagger degree*, the larger the aspect ratio is (the more flattened the CG inclusions is), the better the ability to resist failure and deformation is, which indicates the better ballistic performance.

In the failure processes of all microstructures, microcracks propagate in a region around the central axis. Therefore, along the central axis, the projection length of the adjacent CG inclusions plays a critical role in enhancing the *V*_b_. If the projection length of the adjacent CG inclusions along the central axis is longer, the local ductility of the microstructure will be better along the central axis, which benefits from the better ductility of the CG phase relative to that of the NG phase. The overall ductility is more decisive to enhance their *V*_b_ [32], while the overall strength is more decisive to decrease their *D*_b_ [49,50]. The better local ductility of the microstructure along the central axis will increase the *V*_b_. Obviously, the projection length of the more flattened CG inclusions is shorter, which is unfavorable for the increase in the *V*_b_. However, the microstructures with more flattened CG inclusions achieve higher *V*_b_, which seems to be contrary to the mechanism mentioned above. From the von Mises stress distributions, we find that the more flattened CG inclusions can distribute the stress more effectively in the core region, relieve stress concentration, and retard the microcrack initiation and propagation so that the *V*_b_ increases. Therefore, in terms of *V*_b_, the influence of the stress distributions in the core region arising from the shape of CG inclusions is greater than that of the projection length along the central axis arising from the shape of CG inclusions. Meanwhile, more uniform stress distribution can make the material be utilized more effectively, which is beneficial to enhance the *V*_b_ and decrease the *D*_b_.

**Figure 9:**
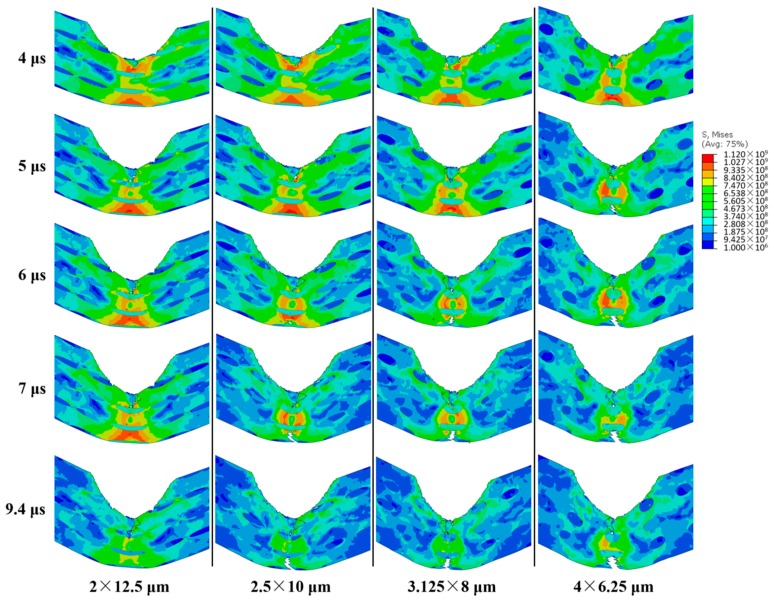
The impact process of the microstructures with the *size* 2 × 12.5, 2.5 × 10, 3.125 × 8, and 4 × 6.25 μm at the 4, 5, 6, 7, and 9.4 μs, when the *stagger degree* is 0, the *f* is 17.45%, and the impact velocity of the bullet is 270 m/s (stress in Pa).

Figure 9 illustrates the impact process of the microstructures with the *size* 2 × 12.5, 2.5 × 10, 3.125 × 8, and 4 × 6.25 μm at 4, 5, 6, 7, and 9.4 μs, when the *stagger degree* is 0, the *f* 17.45%, and the impact velocity of the bullet 270 m/s. From the left to the right in Figure 9, the *V*_b_ of the microstructures decreases gradually. The microstructure with the *size* 2 × 12.5 μm reaches its *D*_b_ at 9.4 μs, while the other three have not reached their *D*_b_. At 4 μs, the stress distribution is the most uniform in the microstructure in the first column, while the most concentrated in the microstructure in the fourth column. At 5 μs, microcracks initiate in the microstructure in the fourth column, which makes the stress distribution more concentrated in the remaining region (the intact materials) around the central axis. The comparison among stress distributions of the four microstructures remains the same as that at 4 μs. At 6 μs, the microcracks initiate in the bottom of the microstructures with the *size* 3.125 × 8 and 2.5 × 10 μm, which makes the stress distribution more concentrated in the remaining region. At 9.4 μs, the microstructure with the *size* 2 × 12.5 μm reaches its *D*_b_ and the microcrack initiates in its upper region only, while more microcracks initiate in their upper and bottom regions of the other three. From the left to the right, the microcracks propagate more and more severely in the microstructures. The propagation of microcracks will weaken the overall stiffness and make the microstructures easy to bend, which will increase their *D*_b_. Therefore, from the left to the right in Figure 9, the *D*_b_ of the microstructure increases significantly.

### 3.3. Effects of the Stagger Degree of Elliptical CG Inclusions

**Figure 10:**
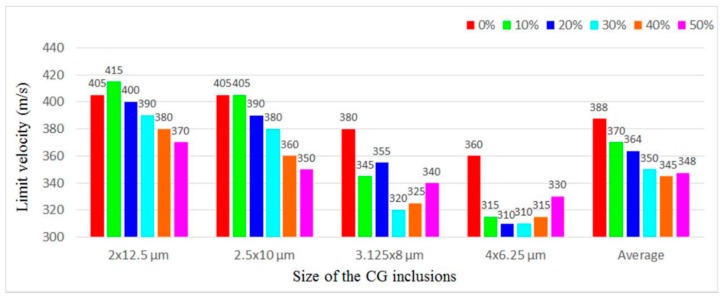
*V*_b_ of the microstructures with different *stagger degree* when the *size* is fixed at different levels and the *f* is 17.45%.

Figure 10 compares *V*_b_ of the microstructures with different *stagger degree* when the *size* is fixed at different levels and the *f* is 17.45%. When the aspect ratio is 6.25 or 4, with the increase in the *stagger degree*, *V*_b_ of the microstructures decreases, which infers that the ability to resist failure can be enhanced with the decrease in the *stagger degree*. The steady drop of *V*_b_ also reflects the relatively stable performance of the microstructures with these aspect ratios of CG inclusions. When the aspect ratio is 2.56 or 1.5625, with the increase in the *stagger degree*, *V*_b_ changes more violently and non-monotonically. In particular, when the aspect ratio is 1.5625, with the *stagger degree* changing from 0 to 10%, *V*_b_ drops tremendously, which infers to the performance instability of the microstructure arising from the distribution of CG inclusions. From the averaged *V*_b_ in Figure 10, we can see that with the *stagger degree* increasing from 0 to 10%, 20%, 30%, 40%, and 50%, the averaged *V*_b_ decreases by 4.64%, 6.19%, 9.79%, 11.08%, and 10.31%, respectively, which indicates that with the increase in the *stagger degree*, the overall trend of *V*_b_ and the ability to resist failure is to decrease.

**Figure 11:**
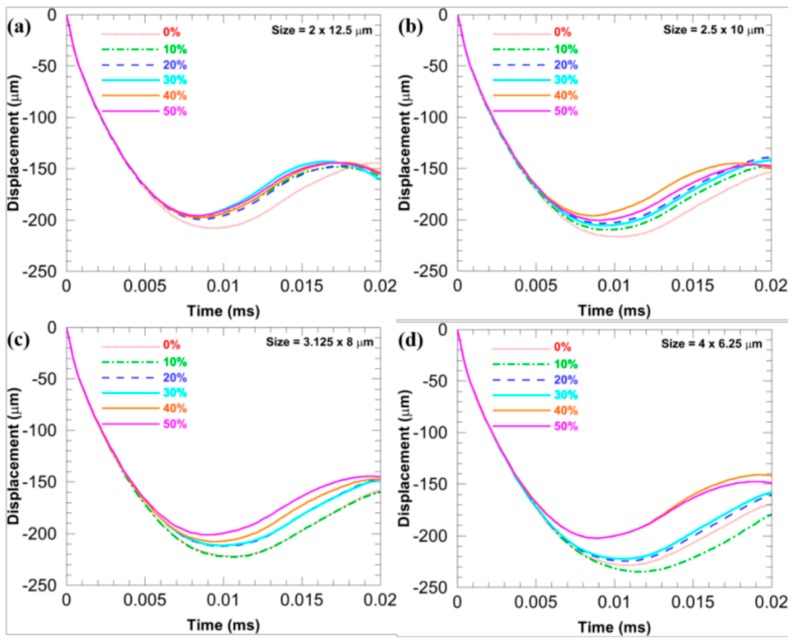
*D*_b_ of the microstructures with different *stagger degree* when the *f* is 17.45% and the *size* is fixed at different levels: (**a**) 2 × 12.5; (**b**) 2.5 × 10; (**c**) 3.125 × 8; and (**d**) 4 × 6.25 μm.

Figure 11 compares *D*_b_ of the microstructures with different *stagger degree* when the *size* is fixed at different levels and the *f* is 17.45%. With the increase in the *stagger degree*, *D*_b_ of the microstructure decreases, which infers that the ability to resist deformation can be enhanced with the increase in the *stagger degree*. This is contrary to the trend of *V*_b_ discussed above. Particularly, in Figure 11d, when the aspect ratio is 1.5625, *D*_b_ of microstructures with the *stagger degree* 40% and 50% are much better than those with the other four *stagger degree*.

As the *stagger degree* increases, *V*_b_ and *D*_b_ of the microstructures exhibit opposite trends in terms of the *stagger degree*—when the *f* is 17.45% and the *size* is fixed at different levels, their ability to resist failure is weakened, while their ability to resist deformation is enhanced. Particularly, when the aspect ratio is small, the increase in the *stagger degree* can significantly enhance the ability to resist deformation of the microstructures.

The microstructures in each sub-figure in Figure 11 have the same aspect ratio, which is different from those in Section 3.2. Therefore, in the core region of each microstructure, there is no difference in the stress distributions arising from the shape of CG inclusions. The difference among the *V*_b_ of each microstructure only arises from that in the stress distributions in the core region arising from the *stagger degree* and the projection length of the CG inclusions along the central axis.

First, with the increase in the *stagger degree*, the projection length of the CG inclusions along the central axis decreases, which means the decrease in local ductility and thus in the *V*_b_.

**Figure 12:**
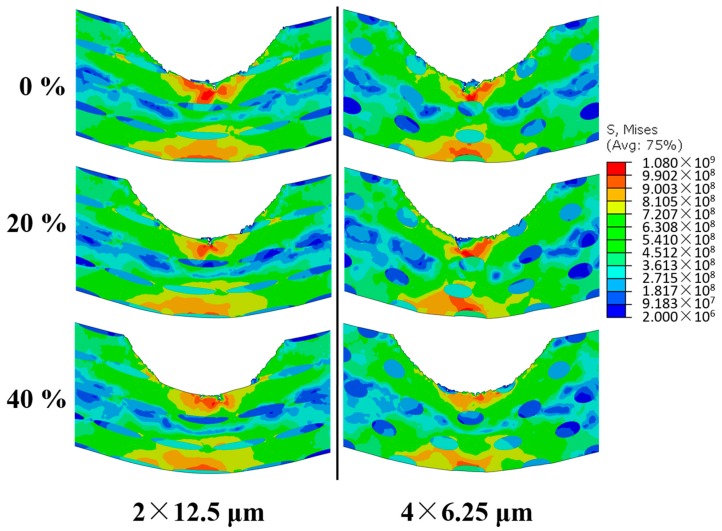
von Mises stress distributions of microstructures with the *f* 17.45% and the initial impact velocity 270 m/s when time is 3 μs after the bullet touches the microstructures (stress in Pa).

Secondly, in terms of the stress distributions in the core region, we find that larger *stagger degree* of CG inclusions can make the stress distribution more uniform, which is conducive to enhancing *V*_b_. On the one hand, since a larger aspect ratio can make the stress distribution more uniform, a larger *stagger degree* of CG inclusions with a larger aspect ratio will not make the stress distribution change evidently. On the other hand, since a smaller aspect ratio can make the stress distribution more concentrated, a larger *stagger degree* of CG inclusions with a smaller aspect ratio can make the stress distribution change evidently, which results in the uncertainty of the change in *V*_b_. Meanwhile, the well-distributed stress caused by staggered CG inclusions can make the overall strength utilized more efficiently, which is beneficial to decrease the *D*_b_. Figure 12 illustrates the von Mises stress distributions of the microstructures with the *f* 17.45% and the initial impact velocity 270 m/s when time is 3 μs after the bullet touches the microstructures. From the left to the right, microstructures have the *size* 2 × 12.5 and 4 × 6.25 μm, respectively; from the top to the bottom, they have the *stagger degree* 0%, 20%, and 40%, respectively. There is nearly no microcrack in all microstructures at 3 μs. The mechanism described above is clearly shown in Figure 12.

According to the mechanisms described above and the data illustrated in Figure 10, we can know that in Section 3.3, in terms of *V*_b_, the effect of the projection length of the CG inclusions along the central axis arising from the *stagger degree* is greater than that of the stress distributions in the core region arising from the *stagger degree*.

In summary, with the increase in the *stagger degree*, both *V*_b_ and *D*_b_ decrease. A larger aspect ratio makes *V*_b_ decrease gradually, while a smaller aspect ratio makes *V*_b_ decrease tremendously and non-monotonically.

### 3.4. Effects of the Volume Fraction of Elliptical CG Inclusions

**Figure 13:**
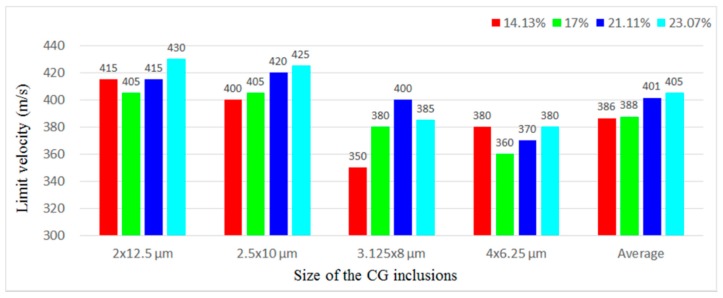
*V*_b_ of four microstructures with different *f* when the base *size* is 2 × 12.5, 2.5 × 10, 3.125 × 8, and 4 × 6.25 μm, while the *stagger degree* is 0.

Figure 13 compares *V*_b_ of four microstructures with different *f* when the base *size* is 2 × 12.5, 2.5 × 10, 3.125 × 8, and 4 × 6.25 μm, while the *stagger degree* is 0. In most cases, when the base *size* and the *stagger degree* is the same, with the *f* increasing, *V*_b_ increases. When the base *size* is 4 × 6.25 μm, with the *f* increasing, *V*_b_ decreases first and then increases. In general, within the scope of this study (14.13% ≤ *f* ≤ 23.07%), the higher the *f* is, the higher the *V*_b_ is, and thus the larger the failure resistance is.

**Figure 14:**
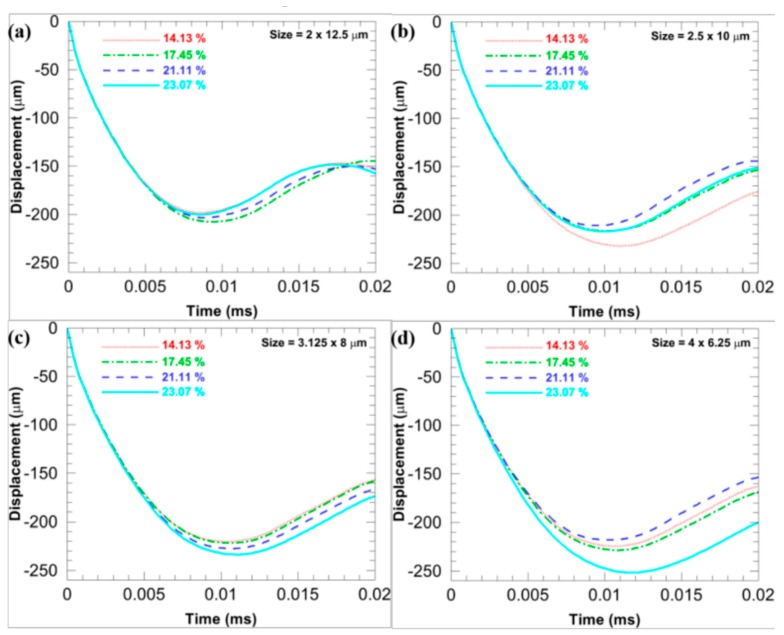
*D*_b_ of four microstructures with different *f* when the base *size* is (**a**) 2 × 12.5; (**b**) 2.5 × 10; (**c**) 3.125 × 8; and (**d**) 4 × 6.25 μm, while the *stagger degree* is 0.

Figure 14 illustrates *D*_b_ of four microstructures with different *f* when the base *size* is 2 × 12.5, 2.5 × 10, 3.125 × 8, and 4 × 6.25 μm, while the *stagger degree* is 0. With the increase in the *f*, no general trend can be found. It is probably because the change in the *f* is not large enough in this study so that the limit displacement does not show a significant trend.

For the sixteen microstructures in Section 3.4, the higher the *f* is, the better the ability to resist failure is. The CG phase plays an active role in this aspect. However, its effect on the ability to resist deformation of the microstructures has not yet been clear.

When the other factors remain unchanged, increasing *f* only will inevitably enhance the overall ductility and decrease the overall strength, which will increase the *V*_b_ and *D*_b_ of the microstructure. The increase in the *f* will also enlarge the projection length of the CG inclusions along the central axis, which will enhance the local ductility of the microstructure along the central axis. This is conducive to increasing the *V*_b_. However, as the increase in *f* is not significant (14.13% to 23.07%), the increase in the *V*_b_ is also limited. Meanwhile, the influence of small change in the *f* is easily interfered by other factors, which makes results complicated and difficult to be analyzed further. This might be the reason for the disorder in the trend of *D*_b_.

## 4. Conclusions and Outlooks

In this paper, we have employed a finite element scheme, based on the strain gradient plasticity model and the Johnson–Cook failure criterion, to systematically investigate the effects of shape (aspect ratio), distribution (*stagger degree*), and volume fraction of elliptical CG inclusions on the ballistic performance of the bimodal NS Cu. The main conclusions can be drawn as follows.

1. The shape of elliptical CG inclusions significantly affects the overall ballistic performance. With a larger aspect ratio of elliptical CG inclusions, the ballistic performance of the microstructure is better.
2. The distribution of elliptical CG inclusions also affects the overall ballistic performance. When the *size* is fixed at a given level and the volume fraction is also fixed, the increase in the *stagger degree* will weaken its ability to resist failure (this is the ductility issue) but will enhance its ability to resist deformation (this is the strength issue).
3. Larger *stagger degree* can weaken the shape effects of elliptical CG inclusions on the overall limit velocity.
4. The projection length of the adjacent CG inclusions along the central axis together with the stress distributions in the core region of the impact has significant effects on the overall ballistic performance.
5. An appropriate increase in the volume fraction of elliptical CG inclusions is helpful to enhance the ability of the microstructure to resist failure under ballistic impact.

The results reported here could shed new light on the application of bimodal NS metals to personal protective equipment.

Although some intrinsic mechanisms such as crack deflection and bridging are quite similar for the meso-scale specimen in this study and the macro-scale specimen in experiments, specific relations between the ballistic performance and the microstructure could be quite different since the size of the meso-scale specimen limits fully development of plastic zone, fracture process zone, or shear band. A scaling law of the ballistic performance on the feature sizes of microstructure, specimen, and bullet deserve further extensive theoretical and computational investigations together with experimental verification.

Furthermore, a recent publication [51] presents a smoothing gradient damage approach for localized failure and tailored to low-order finite elements. An evolving anisotropic nonlocal gradient parameter has been introduced to eliminate spurious damage evolution. To use such a gradient damage model is stimulating in simulating the ballistic impact since it can alleviate the mesh sensitivity encountered in conventional local models.

## Figures and Tables

**Table 1:** The *size* of sixteen microstructures.

*f*	Group I	Group II	Group III	Group IV
14.13%	1.8 × 11.25 μm	2.25 × 9 μm	2.8125 × 7.2 μm	3.6 × 5.625 μm
17.45%	2 × 12.5 μm	2.5 × 10 μm	3.125 × 8 μm	4 × 6.25 μm
21.11%	2.2 × 13.75 μm	2.75 × 11 μm	3.4375 × 8.8 μm	4.4 × 6.875 μm
23.07%	2.3 × 14.375 μm	2.875 × 11.5 μm	3.59375 × 9.2 μm	4.6 × 7.1875 μm
